# Evaluation of the physicochemical and sensory characteristics of gluten-free cookies

**DOI:** 10.3389/fnut.2023.1304117

**Published:** 2024-01-05

**Authors:** Reynaldo J. Silva-Paz, Roxana R. Silva-Lizárraga, Nicodemo C. Jamanca-Gonzales, Amparo Eccoña-Sota

**Affiliations:** ^1^EP Ingeniería de Industrias Alimentarias, Facultad de Ingeniería y Arquitectura, Universidad Peruana Unión, Lima, Peru; ^2^Departamento de Ingeniería – Escuela de Ingeniería en Industrias Alimentarias, Universidad Nacional de Barranca, Lima, Peru

**Keywords:** substitute flours, gluten free, consumers, sensory evaluation, cookies

## Abstract

The increasing prevalence of celiac disease and gluten intolerance has led to an increased demand for gluten-free food products in Peru. The research objective was to develop gluten-free cookies from substitute flours, evaluating their physicochemical and sensory parameters. Eight formulations were developed using 100% broad bean, chickpea, pea, kiwicha, quinoa, lentil, corn, and bean flour. One hundred consumers participated in this evaluation (59% women and 41% men). A completely randomized design (CRD) and a randomized complete block design (RCBD) were used for physicochemical analysis and acceptability, respectively. To describe the sensory characteristics of the cookies, Cochran’s Q-test and correspondence analysis (CA) were performed. From the results obtained, the lentil cookie presented the highest amount of protein and fiber but lower fat and carbohydrate contents compared to the other samples. In terms of color, the corn cookie was the lightest, with greater luminosity (L*), less redness (a*), and greater yellowness (b*). Regarding the sensory analysis, the CATA questions allowed us to form six groups, and the samples with the greatest acceptability were the corn and chickpea cookies, which were rated as “I like them.” Lentil flour crackers are a nutritionally adequate option, and corn flour crackers are highly sensorially acceptable, suggesting commercial opportunities for softer and more flavorful gluten-free products. However, it is crucial to continue researching and developing innovative products to meet changing market demands and offer healthier and more attractive options to consumers.

## 1 Introduction

Several disorders associated with gluten ingestion are now recognized, including celiac disease (CD), intolerance, and gluten allergy (1, 2). In particular, celiac disease is an autoimmune disorder that is triggered in individuals susceptible to the ingestion of gluten from wheat, barley, rye, and others (3). Various studies have identified that approximately 3% of the world population suffers from celiac disease, and until two decades ago it was considered rare, but it has now become widespread worldwide (4). Gluten causes inflammation of the small intestine, atrophy of the villi of the mucosa, and poor absorption of nutrients, and the only treatment for this disease is to have a gluten-free diet for life (5). For this reason, there was a need to develop gluten-free products to meet the demand of celiac consumers who are intolerant or allergic to gluten (6, 7). Food companies that manufacture and supply gluten-free foods and beverages must work with various tools for the development and innovation of foods and decision-making that allow understanding of the success of the product in the market (8).

In recent years, a notable increase in the demand for gluten-free products has been observed, driven by the medical needs of some people suffering from celiac disease and by the conscious choice of consumers who opt for a healthy diet (1, 9). Among these gluten-free products that can be made are cookies, which can be consumed at any time of the day due to their practicality, long shelf life, availability in a presentable format, and having an affordable cost for the consumer (10). The shelf life of the biscuit is prolonged due to its low moisture content, which hinders microbial growth, allowing the product to retain its optimal characteristics for longer, provided it is properly stored (11). The inclusion of flours derived from legumes and pseudocereals in the preparation of cakes, breads, pastas, and cookies represents a technological option that allows us to offer products of nutritional quality, favoring acceptability by the consumer (7, 12, 13). Kaur et al. (6) indicated that the quality of cookies prepared with buckwheat flour and incorporating xanthan gum showed similar sensory profiles to those made with refined wheat flour. On the other hand, Silva et al. (11) mentioned that cookies made with rice and bean flour were rated as innovative products, achieving good acceptability and being recommended for celiac consumers. Similarly, Hamdani et al. (14) reported that cookies prepared with rice and chickpea flour and added gum karaya showed great acceptability by consumers and had a favorable impact on their characterization.

Legumes and pseudocereals have emerged as promising alternative ingredients in the formulation of gluten-free products due to their nutritional profiles, technological functionalities, and unique sensory properties. These ingredients not only offer a rich source of protein, fiber, and other essential nutrients but also present specific characteristics that improve the texture, flavor, and appearance of the final products (15–17). From a technological point of view, they have specific properties, such as the ability to form viscous gels, structural stability, the absence of gluten, a lack of elasticity, and gas retention, which are crucial aspects of achieving a pleasant texture in baked products. When considering consumer acceptance, it is essential to not only address dietary restrictions but also offer alternatives that do not compromise sensory pleasure and the dining experience (18, 19). The benefits of legumes and pseudocereals with the growth of the celiac population have motivated us to propose their exploration as a substitute option for conventional flours in the production of gluten-free products.

A tool that allows understanding of the development and consumption in the market of a product is sensory evaluation, a discipline that encompasses a series of tests and methods to evaluate the perception of food and beverages by the consumer (20, 21). The general acceptability of a product can be evaluated through a hedonic scale that consists of a list of responses with different degrees of satisfaction, where the consumer indicates the response based on their sensory perception (22). There are other methods that allow the description and understanding of the level of enjoyment of the product, such as the check all that applies (CATA) method, where consumers select the attributes that identify the samples evaluated and indicate their acceptability (23). Therefore, the objective of this research was to develop gluten-free cookies from substitute flours and evaluate their physicochemical and sensory parameters.

## 2 Materials and methods

### 2.1 Ingredients

Different substitute flours of lentils, peas, common beans, white corn, chickpeas, broad beans, kiwicha, and white quinoa, obtained in the central market of Lima, were used, in addition to other ingredients such as butter, brown sugar, sodium bicarbonate, egg, and water, where the percentages used for the different formulations are shown.

The formulation of the cookies consisted of G1 (broad bean, 53.80, 8.00, 16.10, 0.50, 10.80, and 10.80% of flour, butter, brown sugar, baking soda sodium, egg, and water, respectively), G2 (chickpea, 56.80, 8.50, 17.0, 0.60, 11.40, and 5.70%), G3 (pea, 56.80, 8.50, 17.00, 0.60, 11.40, and 5.70%), G4 (kiwicha, 55.20, 8.30, 16.60, 0.60, 11.00, and 8.30%), G5 (quinoa, 55.20, 8.30, 16.60, 0.60, 11.00, and 8.30%), G6 (lentil, 55.20, 8.30, 16.60, 0.60, 11.00, and 8.30%), G7 (corn, 53.80, 8.00, 16.10, 0.50, 10.80, and 10.80), and G8 (common bean, 56.80, 8.50, 17.00, 0.60, 11.40, and 5.70%,). All formulations were designed according to the proposal by the American Association for Clinical Chemistry (AACC) (24).

### 2.2 Gluten-free cookie-making process

To prepare the cookies, we followed the procedure outlined by Huatuco et al. (25), with some adjustments. Initially, we measured all the ingredients based on the formulations detailed in Section 2.1. For the creaming process, we combined butter and brown sugar in a KitchenAid mixer (Model: Artisan, United States) at 6 rpm for 5 min until a uniform mixture was achieved. Eggs were then added and beaten at 4 rpm for 5 min to form a smooth, creamy emulsion. Subsequently, the substitute flour was manually mixed with sodium bicarbonate in a stainless-steel container. The cream mixture was then added to this new blend and mixed for an additional 5 min. Gradually, water was incorporated until a homogeneous dough was attained. The dough was rolled to a thickness of 5 mm and molded to a diameter of 40 mm. The resulting products were baked in a rotary oven (Brand: Nova, Model: Max 600, Peru) at 140°C for 10 min, followed by cooling at room temperature for 20 min. They were then packaged in polyethylene bags and hermetically sealed. Finally, the cookies were stored at room temperature for subsequent physical, chemical, and sensory analyses.

### 2.3 Physicochemical analysis

#### 2.3.1 Nutritional composition analysis

Moisture and ash contents were determined according to the AOAC analysis method (26), while crude fat, crude protein, and crude fiber were determined by the AACC analysis method (27), and the amount of carbohydrates was calculated by difference (28).

#### 2.3.2 Color analysis

The color was determined using a portable colorimeter (3nh brand, model Nh310, China). The cookies were placed in direct contact to measure the color of the surface. This analysis was performed in triplicate using the CIEL*a*b* system. The parameters to be measured were L* (brightness) [(0) black / (100) white], a* [(+) red / (−) green], and b* [(+) yellow / (−) blue] (11, 22). In addition, the whiteness index (WI) was determined, WI= $100-\sqrt{{\left(100-L*\right)}^{2}+a{*}^{2}+b{*}^{2}\;}$ (29) and the browning index (BI) (30), BI = (100*((x-0.31)/0.17), where x is equal, x = (a + 1.75 L*)/(5.645 L* + a* - 3.012b*) using the parameters L*, a*, and b*. The color parameters of the control sample to quantify the ΔE were L* = 76.26 ± 0.24, a* = 4.42 ± 0.19, and b* = 40.45 ± 0.28. To determine the ΔE = $\sqrt{{\left(L*-Lo*\right)}^{2}+{\left(a*-ao*\right)}^{2}+{\left(b*-\mathrm{bo}*\right)}^{2}\;}$ , where Lo *, a*, and b* correspond to the values of the control sample and L*, a*, and b* are the data of each sample.

### 2.4 Sensory analysis

#### 2.4.1 Consumers

The evaluators were recruited from the Faculty of Engineering and Architecture of the Universidad Peruana Unión, with a total of 100 consumers, of whom 59% were women and 41% were men (aged 24 ± 6 years). Their participation was voluntary, and the study was carried out with informed consent approved by the Ethics Committee of the Faculty of Engineering and Architecture of the Universidad Peruana Unión (N° 2022-CEFIA-0006).

#### 2.4.2 Check all that apply (CATA)

All participants received eight cookies randomly, coded with three digits, and delivered monadically. Previously, consumers gave informed consent to participate in the sensory tests and were provided with general instructions on the CATA methodology. Then, the evaluation sheet was provided with 13 sensory attributes, of which 7 described the texture (sticky, soft, crunchy, brittle, hard, greasy, and porous), 3 described the taste (bitter, sweet, and strange taste), 2 described the appearance (light color and dark color), and 1 described the aroma (strange smell). These terms were selected from previous studies (31–33). For the sensory test, participants were asked to select the terms they considered appropriate to describe the samples (34, 35). The samples were evaluated in a single session of approximately 30 min in the laboratory of the Food Science Research Center (CICAL) of the Universidad Peruana Unión. Participants were instructed to drink table water between each sample to cleanse the palate.

#### 2.4.3 Overall liking

**Figure 1:**
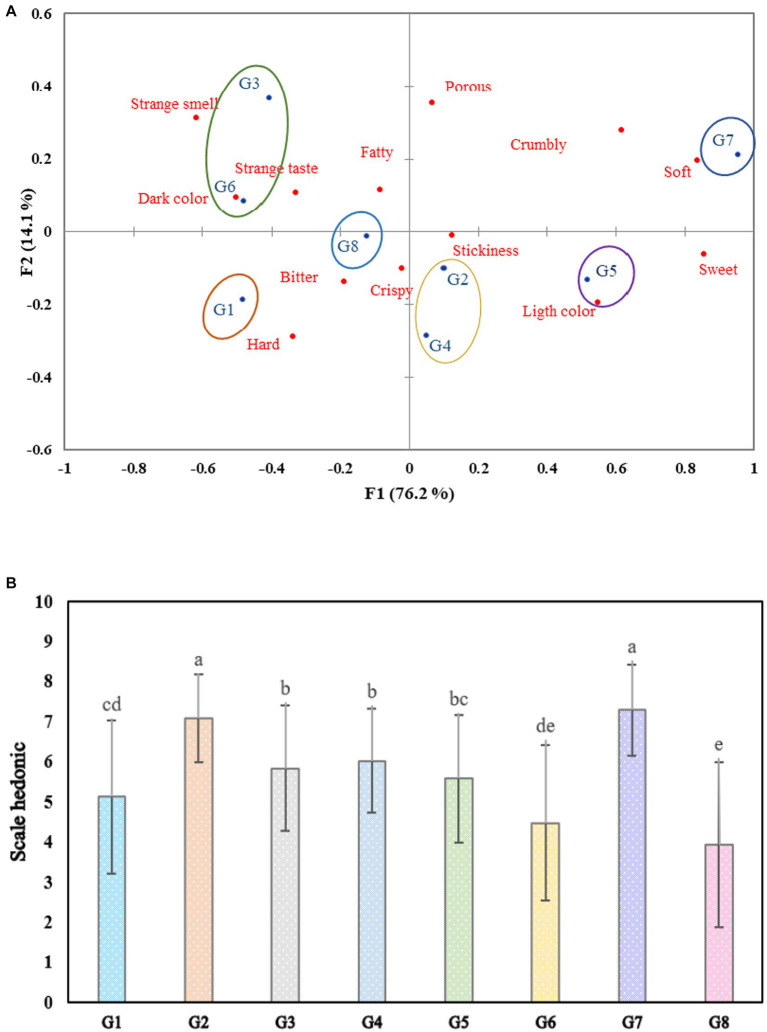
Map resulting from perception correspondence analysis (CA) using the CATA method **(A)** and cookie acceptability using a 9-point hedonic scale **(B)**. (Cookie, G1: broad bean, G2: chickpea, G3: pea, G4: kiwicha, G5: quinoa, G6: lentil, G7: corn, and G8: common bean).

For the liking test, a 9-point hedonic scale was used, with the highest score being I like it very much (9 points) and the lowest being I dislike it a lot (1 point). Consumers were instructed to rate the samples according to their perception, as well as to drink table water between each sample to minimize the carryover effect and influence the evaluation from the first to the last sample (11, 36). Figure 1 describes the stages of the research process, from the use of flour to the preparation of cookies to the physicochemical and sensory tests carried out.

### 2.5 Statistical analysis

For the data of the physicochemical analysis, a completely randomized design (CRD) was applied (25, 37), and for the general acceptability, a completely randomized block design (RCBD) (38, 39) was carried out to meet the assumptions of normality and homogeneity of variance. When evaluating the analysis of variance (ANOVA) and identifying if there were significant differences, and in the case of finding significance (*p* < 0.05), the Tukey test was carried out at a confidence level of 95% (11, 40) using the statistical software R.

Correspondence analysis (CA) was applied to the data obtained by the check all that apply (CATA) method to obtain the association map between the samples and the sensory attributes (41, 42). In addition to Cochran’s Q-test to identify significant differences (*p* < 0.05) between the samples and the constancy of use of each attribute (43, 44), the statistical program XLSTAT 2023 was used for these analyses.

## 3 Results

### 3.1 Physico-chemical analysis

#### 3.1.1 Nutritional composition analysis

The proximal composition of the cookies is shown in Table 1, expressed in percentages (%). G7 and G5 presented a higher moisture value and did not show significant differences (*p* > 0.05) between both samples. Similarly, it was observed that G4 acquired a smaller amount of ashes compared to G8, which was statistically superior (*p* < 0.05). Regarding the fat content, G2 registered a higher content than the rest of the cookies. According to the amount of fiber, the highest values were G3, G6, and G8; these samples did not show significant differences (*p* > 0.05). A reduced content of protein was presented by G7; on the contrary, G6 obtained the highest amount, showing significant differences (*p* < 0.05). On the other hand, G7 presented a higher carbohydrate content, although similar to G1, G3, G4, and G5 (*p* > 0.05).

**Table 1:** Proximate composition of biscuits on dry base (g/100 g).

Sample	Moisture content (%)	Ash (%)	Fat (%)	Protein (%)	Fiber (%)	Carbohydrates (%)
G1: Broad bean	5.17 ± 0.07^c^	2.31 ± 0.19^abc^	12.10 ± 0.13^b^	10.60 ± 0.33^d^	2.75 ± 0.30^a^	67.10 ± 0.49^ab^
G2: Chickpea	5.20 ± 0.08^c^	2.89 ± 0.03^a^	13.50 ± 0.05^a^	10.30 ± 0.05^d^	2.63 ± 0.47^a^	65.50 ± 0.46^cd^
G3: Pea	5.45 ± 0.27^b^	2.46 ± 0.40^ab^	9.58 ± 0.12^de^	12.60 ± 0.41^b^	3.46 ± 0.18^a^	66.50 ± 0.32^ab^
G4: Kiwicha	5.37 ± 0.10^bc^	1.69 ± 0.09^c^	10.40 ± 0.08^cd^	12.20 ± 0.09^bc^	2.67 ± 0.19^a^	67.70 ± 0.20^ab^
G5: Quinoa	5.96 ± 0.19^ab^	1.91 ± 0.06^bc^	10.50 ± 0.45^cd^	11.50 ± 0.17^c^	2.72 ± 0.04^a^	67.50 ± 0.07^ab^
G6: Lentil	5.54 ± 0.16^b^	2.62 ± 0.11^a^	9.19 ± 0.09^e^	13.80 ± 0.11^a^	3.60 ± 0.16^a^	65.20 ± 0.09^cd^
G7: Corn	6.13 ± 0.04^a^	2.34 ± 0.08^abc^	11.20 ± 0.40^bc^	8.49 ± 0.16^e^	3.31 ± 0.33^a^	68.60 ± 0.46^a^
G8: Common Bean	5.39 ± 0.26^bc^	2.94 ± 0.05^a^	10.30 ± 0.26^cd^	12.80 ± 0.13^b^	3.45 ± 0.06^a^	65.10 ± 0.42^cd^

^a, b, c, d, e^ Different superscripts represent significant differences (*p* < 0.05), according to the Tukey method.

#### 3.1.2 Color analysis

Table 2 shows the color parameters expressed in dimensionless units; this is an important property for the acceptance of cookies by consumers. Sample G7 was the sample with the highest luminosity, while G6 obtained the lowest value of coloration (dark). The samples presented significant differences (*p* < 0.05). In the a* parameter, the samples were between the values of 15.4 ± 0.32 and 30.4 ± 0.05, showing little redness. While in the b* parameter, the values ranged between 45 and 56, indicating greater yellowing. The whiteness index of sample G7 is significantly different from the other samples, and G8 showed lower whiteness. For the browning index, an inverse behavior was observed for these samples. Samples G7 and G2 showed lower values of delta E; that is, they did not show large differences with the control sample.

**Table 2:** Color parameters using the CIE Lab system for cookies.

Sample	L* (0) black/(100) white	a* (+) red/(−) green	b* (+) yellow/(−) blue	Whiteness index (WI)	Browning index (BI)	ΔE
G1: Broad bean	48.7 ± 0.34^e^	18.4 ± 0.43^de^	47.1 ± 0.08^bc^	27.9 ± 0.41^e^	220.0 ± 3.99^d^	31.6 ± 0.51^d^
G2: Chickpea	55.2 ± 0.12^b^	17.8 ± 0.33^e^	47.9 ± 0.36^b^	38.7 ± 0.44^b^	135.6 ± 2.13^f^	18.3 ± 0.45^g^
G3: Pea	46.1 ± 0.45^f^	25.5 ± 0.32^b^	56.9 ± 0.41^a^	17.6 ± 0.67^g^	364.8 ± 14.2^b^	40.3 ± 0.67^b^
G4: Kiwicha	54.8 ± 0.39^c^	19.3 ± 0.04^d^	45.6 ± 0.26^de^	33.0 ± 0.42^c^	171.5 ± 3.37^e^	26.6 ± 0.34^f^
G5: Quinoa	51.0 ± 0.38^d^	18.9 ± 0.33^d^	46.3 ± 0.22^cd^	29.9 ± 0.21^d^	198.0 ± 1.46^de^	29.7 ± 0.44^e^
G6: Lentil	43.2 ± 0.40^g^	20.9 ± 0.08^c^	47.3 ± 0.18^bc^	23.2 ± 0.42^f^	281.5 ± 7.25^c^	37.5 ± 0.42^c^
G7: Corn	80.0 ± 0.34^a^	15.4 ± 0.32^f^	45.1 ± 0.10^e^	48.3 ± 0.05^a^	93.7 ± 0.01^g^	12.5 ± 0.42^h^
G8: Common Bean	44.0 ± 0.35^g^	30.4 ± 0.05^a^	57.4 ± 0.45^a^	14.2 ± 0.55^h^	415.7 ± 15.40^a^	44.8 ± 0.45^a^

^a, b, c, d, e, f, g, h^ Different superscripts present significant differences (*p* < 0.05), according to the Tukey method.

### 3.2 Sensory analysis

#### 3.2.1 Consumers

In this study, 100 consumers participated, of whom 59% were women and 41% were men (aged 24 ± 6 years). Of all the women, 54% were from the coast, 27% were from the mountains, and 19% were from the jungle. In addition, 63% preferred the chocolate flavor, 13% preferred vanilla, 14% preferred Andean grains, and 10% preferred salty; additionally, it was found that 67% eat cookies frequently, 19% sometimes, and 14% eat very few cookies. Of the participants, 79% were from the “Coast” region, 15% from the “Sierra,” and 6% from the “Selva.” Similarly, it was observed that 62% prefer the chocolate flavor, 20% prefer vanilla, 9% prefer Andean grains, and 9% prefer salty. On the other hand, it was found that 65% eat cookies frequently, 23% sometimes, and 12% eat very few cookies.

#### 3.2.2 Check all that apply (CATA) method and overall acceptability

Cochran’s Q-test shown in Table 3 shows that consumers found significant differences (*p* < 0.05) in 11 of the 13 attributes evaluated in the CATA questions, so the use of the CATA method allows the description of similar and different characteristics of the cookies. Samples G4, G5, and G7 were similar to each other in the attributes of “dark color” and “strange smell,” as well as G1, G3, G6, and G8, with G3 and G6 being considered more frequently as having a dark and strange smell. Regarding the attributes “porous” and “strange taste,” samples G1, G2, G4, G5, G6, G7, and G8 showed similarity, presenting less porosity and a strange flavor, unlike G3. Regarding the “bitter” attribute, samples G1 and G8 were similar to each other, although they were described as bitter, unlike G2, G3, G4, G5, and G7. Samples G1, G3, G6, and G8 were similar to each other in the “light color” attribute, as were G2, G5, and G7, with G5 being the sample characterized as light. Regarding the attributes “soft,” “crumbly,” and “sweet,” samples G1, G2, G3, G4, G6, and G8 were similar to each other, differing from G7, which was described as soft, crumbly, and sweet. For the “hard” attribute, samples G1, G4, G6, and G8 showed similarity, as did G2, G3, and G5, with G1 being the sample most frequently mentioned as hard, unlike the others.

**Table 3:** Cochran’s Q-test of the attributes evaluated by consumers.

Attribute	*p*-value	G1	G2	G3	G4	G5	G6	G7	G8
Dark color	0.00	0.68^c^	0.35^b^	0.70^c^	0.27^ab^	0.10^ab^	0.78^c^	0.07^a^	0.69^c^
Porous	0.00	0.13^a^	0.12^a^	0.40^b^	0.13^a^	0.21^a^	0.20^a^	0.28^ab^	0.16^a^
Bitter	0.00	0.51^c^	0.20^ab^	0.25^ab^	0.26^ab^	0.24^ab^	0.29^b^	0.16^a^	0.56^c^
Light color	0.00	0.17^ab^	0.49^cd^	0.09^a^	0.42^c^	0.63^d^	0.11^a^	0.52^cd^	0.20^ab^
Fatty	0.10	0.02^a^	0.07^a^	0.11^a^	0.06^a^	0.07^a^	0.06^a^	0.02^a^	0.04^a^
Soft	0.00	0.02^a^	0.18^ab^	0.10^ab^	0.11^ab^	0.23^b^	0.05^a^	0.51^c^	0.11^ab^
Stickiness	0.77	0.05^a^	0.10^a^	0.07^a^	0.05^a^	0.09^a^	0.05^a^	0.06^a^	0.06^a^
Crumbly	0.00	0.02^a^	0.21^ab^	0.17^ab^	0.09^a^	0.28^b^	0.15^ab^	0.51^c^	0.21^ab^
Hard	0.00	0.85^d^	0.39^b^	0.41^b^	0.66^cd^	0.36^b^	0.70^cd^	0.05^a^	0.68^cd^
Strange taste	0.00	0.24^ab^	0.18^ab^	0.35^b^	0.20^ab^	0.09^a^	0.26^ab^	0.09^a^	0.23^ab^
Sweet	0.00	0.09^a^	0.23^ab^	0.05^a^	0.29^ab^	0.44^c^	0.05^a^	0.65^d^	0.12^ab^
Strange smell	0.00	0.38^cd^	0.27^b^	0.56^d^	0.16^ab^	0.01^a^	0.45^cd^	0.04^a^	0.40^cd^
Crispy	0.00	0.38^ab^	0.48^ab^	0.35^ab^	0.43^ab^	0.40^ab^	0.38^ab^	0.28^a^	0.52^b^

^a, b, c, d, e^ Different superscripts represent significant differences (*p* < 0.05), according to Cochran’s Q-test.

Figure 1A shows eight cookie samples and the sensory attributes used to describe them in the first two dimensions. Where six defined subgroups are observed, the first group formed by G1 was characterized as hard. The second group, which is G3 and G6, had the characteristics of dark color and a strange smell, while G8, which represents the third group, was described as bitter and crunchy. The fourth group, made up of G2 and G4, was determined as crispy and adhesive. The fifth group, which is G5, was characterized by a light color. Finally, the sixth group formed by G7 was rated as soft. In Figure 1B, we can find that G7 and G2 reached the highest scores by not registering significant differences (*p* > 0.05) between both samples, being moderately evaluated as I like. However, G8 was the least accepted and evaluated as “Dislike moderately.” This could be because it was characterized as bitter.

## 4 Discussion

### 4.1 Physicochemical analysis

#### 4.1.1 Nutritional composition analysis

According to the results obtained from the proximal analysis in Table 1, G6 was lower than that reported by Gómez et al. (45) in biscuits partially substituted with legume flours. This could be because the lentil flour was previously induced to reach a moisture content of 10%. Soler et al. (46) found a humidity of 1.11 ± 0.05 in biscuits based on 100% bean flour, although this result is lower than that found in G8 of the present study. This difference could be due to the variety of the ingredients.

On the other hand, the highest ash content was obtained by G8, as well as the biscuit made with bean flour in the study carried out by Gómez et al. (45) in biscuits partially substituted with legume flour and as reported by Soler et al. (46) in the cookie with the 100% bean formulation. Millar et al. (47) mentioned that the high content of ashes in legume flours increases the intake of minerals in the diet. In another order of ideas, G2 presented the highest fat value compared to the rest of the cookies, as indicated by Gómez et al. (45) in the biscuit made with chickpea flour in the partial substitution of legume flour. Similarly, Foschia et al. (12) mentioned that chickpea flour has the highest lipid content compared to other legumes. Silva et al. (11) reported fat values ranging from 9.83 ± 0.98 to 11.61 ± 1.10 in crackers made with rice and beans, with the result obtained in G8 being within these values. In terms of fiber, G5 obtained a value of 3.45 ± 0.06, whereas Huatuco et al. (25) found values ranging from 6.3 to 11.3 in cookies made with wheat flour, granadilla, and quinoa; these results were superior to this research. On the other hand, G8 was similar to what was found by Soler et al. (46) in formulation F100 (3.38 ± 0.04) of the cookie made with 100% bean flour. A high fiber content is essential for celiac consumers since gluten-free products generally have a low fiber content, and their intake can induce obesity and other health risks (11). The protein content in G6 was similar to that reported by Gómez et al. (45) in biscuits partially substituted with legume flour, reporting a value of 14.3 ± 0.4% for biscuits made with lentil flour. Regarding the proteins in G8, it was higher than that reported by Silva et al. (11) in crackers made with rice and beans that obtained values from 7.99 ± 0.23 to 10.10 ± 0.48%; this may be due to the fact that some ingredients had a cooking process before. On the other hand, the amount of protein in G7 was lower than the rest of the cookies; this is due to the fact that corn flour has a low protein content (9). The carbohydrate content of G8 differed from that reported by Soler et al. (46) in the cookie made with 100% bean flour, which could be attributed to the type of grain and the method used to obtain the flour. The cookies made by Gómez et al. (45), the one substituted with chickpea flour, obtained a carbohydrate content of 59.5%, which was lower than that reported in G2. Foschia et al. (12) mentioned that, in general, the total content of carbohydrates in legumes constitutes between 45 and 66% of the dry weight.

#### 4.1.2 Color analysis

The results of the chromatic parameters are shown in Table 2. L* (80.0 ± 0.34) in G7 was similar to that found by Gutiérrez et al. (48) in corn crackers, and different types of starch in treatment 1 were L* (84.48 ± 1), but the values of a* and b* differed. On the other hand, G8 presented a lower L* (44.0 ± 0.35) but a higher a* (15.4 ± 0.32) and b* (45.1 ± 0.10) compared to L* (91.13 ± 1.35), a* (−0.28 ± 0.02), and b* (6.16 ± 0.15) in crackers made from rice and beans, as reported by Silva et al. (11). This difference may be due to the fact that polished rice contains mostly starch. In another study by Hamdani et al. (14), there were higher values in L* (55 ± 1 to 56 ± 3) and lower values in a* (2 ± 0.3 to 4 ± 1) and b* (32 ± 0.2 to 35 ± 1) in cookies made with rice, chickpea, and gum flour compared to G2, which was found to be 55.2 ± 0.12 in L*, 17.8 ± 0.33 in a*, and 47.9 ± 0.36 in b*. This could be due to the speed at which the Maillard reaction occurs since it varies according to the type of sugar.

### 4.2 Sensory analysis

#### 4.2.1 Check all that apply (CATA) method

In Table 3, consumers differentiated 11 of the 13 sensory attributes, similar to the research carried out by Rocha et al. (49) in sweet cookies, where they identified significant differences in 15 of the 21 sensory descriptors. They suggest that this method allows samples to be distinguished according to the perception of the evaluators.

The graphic representation of the samples and sensory attributes in Figure 1A explains 90.3% of the total variation, which agrees with Pramudya and Seo (43) and Rocha et al. (49), who presented total variations of 92.95 and 97.01%, respectively, where they illustrate the associations between the samples and the sensory descriptors in the first two dimensions of the correspondence analysis (CA).

The G7 sample based on corn flour was considered the softest, most crumbly, and sweetest. This is because starch has the functionality of improving the texture, decreasing the hardness, and increasing the characteristic flexibility of the products baked (48, 50).

#### 4.2.2 Overall acceptability

The samples with the highest acceptability were based on corn flour (G7) and chickpea (G2), while the least admissible one was made with bean flour (G8), as observed in Figure 1B. This result was similar to what was reported by Gómez et al. (45) when evaluating the effect of the partial substitution with legume flours. In another study, Gutiérrez et al. (48) found that treatment 3 (90% corn flour and 10% starch) obtained the highest acceptability from evaluators for corn crackers and different types of starch. Similarly, Hamdani et al. (14) mentioned that cookies made with rice flour and chickpea-added karaya gum were the most accepted by consumers because they showed the highest ratings in appearance, mouthfeel, flavor, and mainly texture, helping to reduce the hardness of cookies.

It is important to highlight the increasing demand for gluten-free diets due to the prevalence of celiac disease and gluten sensitivity. The research allowed us to develop specific formulations that improve the texture, flavor, and quality of gluten-free cookies, generating a direct impact on the formulation of commercial products and consumer preference. Furthermore, by addressing sensory attributes, it allows for the improvement of marketing and positioning strategies for gluten-free products, providing the industry with valuable information to adapt to constantly evolving market demands. This approach will help to highlight the practical importance of research and its contribution to knowledge in the field of gluten-free products.

## 5 Conclusion

Gluten-free cookies were developed, with significant differences in the physicochemical, colorimetric, and sensory parameters. Of the different formulations made, the lentil flour cookie had a higher protein and fiber content with reduced levels of fat and carbohydrates, which distinguished it from other cookies. The corn and chickpea flour cookies obtained the highest acceptability scores compared to the rest of the cookies, being described as soft, crunchy, and sticky. These findings highlight the viability of gluten-free cookies as an accessible and marketable option, especially aimed at people with celiac disease, gluten intolerant people, and those seeking a healthy diet. The research not only offers a solution to the dietary needs of this demographic but also presents a sensory-appealing product. However, it is recognized that there is a need for future research to delve into the optimization of the formulation, shelf life, and production quality to further improve commercialization and provide a more complete and robust alternative in the gluten-free product market.

## Data availability statement

The original contributions presented in the study are included in the article/supplementary material, further inquiries can be directed to the corresponding author.

## Ethics statement

This study was approved by Ethics Committee of Facultad de Ingeniería y Arquitectura - Universidad Peruana Unión (N° 2022-CEFIA-0006).

## Author contributions

RS-P: Conceptualization, Methodology, Software, Writing – original draft, Writing – review & editing. RS-L: Data curation, Investigation, Methodology, Writing – original draft. NJ-G: Conceptualization, Data curation, Formal analysis, Writing – original draft. AE-S: Funding acquisition, Methodology, Project administration, Supervision, Writing – review & editing.
